# Characteristics of Graves' disease in children and adolescents in Nanjing: A retrospective investigation study

**DOI:** 10.3389/fpubh.2022.993733

**Published:** 2022-10-11

**Authors:** Hang Xie, Dandan Chen, Ju Zhang, Ruize Yang, Wei Gu, Xu Wang

**Affiliations:** ^1^Office of Clinical Research, Children's Hospital of Nanjing Medical University, Nanjing, China; ^2^Department of Endocrinology, Children's Hospital of Nanjing Medical University, Nanjing, China; ^3^Department of Public Health, Children's Hospital of Nanjing Medical University, Nanjing, China

**Keywords:** Graves' disease, hyperthyroidism, Nanjing, children and adolescents, clinical characteristic

## Abstract

**Objective:**

This cross-sectional study analyzed the clinical characteristics of newly diagnosed Graves' disease (GD) in children and adolescents to provide clinical evidence for the early diagnosis of GD.

**Method:**

From 2013 to 2019, information on children and adolescents with newly diagnosed GD admitted to the Department of Endocrinology, Children's Hospital of Nanjing Medical University, was collected, including clinical features and laboratory tests. The data were summarized and statistically analyzed.

**Result:**

This study included 204 cases of newly diagnosed GD, with 158 females and 46 males. The average age at initial diagnosis was 8.9 ± 2.9 years. A total of 132 cases (64.7%) had symptoms before puberty, and 72 cases (35.3%) had symptoms during puberty. Goiter was detected in 193 cases (94.6%). There were 140 cases (68.6%) of exophthalmos, and 21.4% (30/140) were infiltrative. At initial diagnosis, 10 cases (4.9%) reported leukopenia, 18 cases (8.8%) reported neutropenia, and 15 cases (7.4%) reported mild anemia. There was 1 (0.5%) case of thrombocytopenia and 1 (0.5%) case of agranulocytosis. Fifty-four cases (26.5%) had impaired liver function.

**Conclusion:**

GD is more common in female children and adolescents. Parents may ignore their children's hypermetabolic symptoms in the early stages of GD. Routine blood and liver function tests are recommended at initial diagnosis to exclude abnormal hemogram and liver function.

## Introduction

Graves' disease (GD) is an autoimmune thyroid disease (ATID) that accounts for ~95% of the causes of thyrotoxicosis in children and adolescents (1). GD is caused by the combined effects of multiple genetic, environmental, and immune factors. The incidence of GD in children and adolescents is ~0.02% (2). Significantly more female cases than male cases have been reported, and most of these cases were adolescents (3). The incidence of GD in adolescents has been increasing annually (4, 5).

Characteristics of GD include hypermetabolic syndrome, goiter, and exophthalmos. It also influences the growth and puberty development of children and adolescents (6, 7). Unlike GD in adult populations, goiter in children and adolescents is generally mild, and pretibial myxedema and hyperthyroidism crises are rarely reported (8). Notably, the clinical manifestations are diverse, and some children and adolescents present to the hospital with atypical symptoms, such as vomiting, fainting, and headache (9). Emotional changes and hypermetabolic features are easily ignored, and it may take several years from the onset of the disease to achieve a definitive diagnosis (10). The etiology of a few children and adolescents is unknown (11).

GD affects multiple organ systems, including the cardiovascular, gastrointestinal, and hepatic systems (12). Liver dysfunction is common in adult GD patients. Most patients only have abnormal liver function indices, with no obvious symptoms. However, a few patients experience serious liver damage or liver failure (13–16). Liver dysfunction in children and adolescents with GD was also reported (17, 18). However, the sample size limits relevant studies in the pediatric population. In addition, abnormal peripheral blood cells, such as leukopenia or granulocytopenia, also require concern in the GD pediatric population (19).

Nowadays, studies on GD in children and adolescents are increasing (1, 20–22), while only a few related studies have been performed in China (9, 11, 23). The current study was a retrospective analysis to summarize the signs, symptoms, thyroid hormone levels, and abnormal liver function and hemogram of GD children and adolescents. The results provide evidence on liver function, hemogram, and clinical decisions in GD children and adolescents.

## Methods

### Population

The clinical data of all children and adolescents with newly diagnosed and untreated GD were collected. The Medical Ethics Committee of the Children's Hospital of Nanjing Medical University approved this study (No: 2022011003-1). The following inclusion criteria were used for this study:

(1) From January 2013 to August 2019, children and adolescents with newly diagnosed and untreated GD who were admitted to the Department of Endocrinology, Children's Hospital of Nanjing Medical University.
(2) Children and adolescents who met the diagnostic criteria in the Guidelines for Diagnosis and Treatment of Thyroid Diseases in China, which was issued by the Chinese Medical Association (7): (i) clinical symptoms and signs of hyperthyroidism; (ii) goiter confirmed by palpation or thyroid ultrasound; (iii) decreased levels of serum thyroid-stimulating hormone (TSH) and increased levels of thyroid hormone (TH); (iv) proptosis and other infiltrative ocular signs; (v) pretibial myxedema; and (vi) positive thyrotropin receptor antibodies (TRAb) or thyroid-stimulating antibodies (TSAb). The first three conditions are prerequisites, and the remaining three are auxiliary conditions.

The exclusion criteria included (1) thyroid tumors, (2) Hashimoto's thyroiditis, (3) neonatal GD, (4) drug-induced secondary hyperthyroidism, and (5) other diseases that did not match the signs of GD thyroid ultrasound findings.

### Data collection

General information, including sex, height, body weight, heart rate, family history of thyroid disease, births, puberty development, and initial chief complaints, was collected. Symptoms and signs, such as exophthalmos, tremor, and goiter, were assessed and recorded. Correlative laboratory tests, including routine blood examination, serum biochemical levels, thyroid function, and thyroid-related antibodies, were also collected. An automatic biochemical analyzer for biochemistry, chemiluminescence method for thyroidal function and blood cell analyzer for routine blood tests were used in the sampling analysis. Enzyme-linked immunosorbent assays were used for TRAb determination.

### Standards of division

(1) Degrees of goiters (10): (i) level I: non-visible but palpable enlargement, (ii) level II: palpable and visible enlargement that did not exceed the outer edge of sternocleidomastoid muscle, (iii) level III: obvious enlargement that reached the outer edge of sternocleidomastoid muscle.
(2) Assessment of pubertal development (6): the secondary sexual characteristics of patients were evaluated according to the Tanner staging criteria.
(3) Classifications of exophthalmos (24): (i) simple exophthalmos: slight proptosis of the eyeballs, broadening palpebral fissure and reduced blinking, (ii) infiltrating exophthalmos: significant proptosis of the eyeballs. Patients might have pain, photophobia, lacrimation, diplopia, strabismus, impaired vision and foreign body sensation. Physical examination revealed swollen eyelids, edematous and congestive conjunctiva, and restricted movement of the eyeballs. Fixed eyeballs, incomplete closure of the eyelid, corneal ulcers caused by exposed corneas, panophthalmitis, and blindness are found in severe cases.
(4) Classification for severity of hyperthyroidism (22): (i) mild hyperthyroidism: normal upper limit < free thyroxine (FT4) ≤ 1.5 times normal upper limit, (ii) moderate hyperthyroidism: 1.5 times the normal upper limit < FT4 ≤ 2.5 times the normal upper limit, And (iii) severe hyperthyroidism: FT4 > 2.5 times the normal upper limit.

### Statistical analysis

Numerical data with skewed distributions are expressed as medians and interquartile ranges and were analyzed using the Wilcoxon rank sum test. Categorical data are shown as percentages and were analyzed using the chi-squared test. Since FT4, TSH, TRAb, thyroglobulin antibody (TGAb), and thyroid peroxidase antibody (TPOAb) concentrations were not normally distributed, Spearman rank correlation analysis was used for bivariate correlation analysis. All statistical analyses were performed using SPSS 26.0 statistical software (IBM, USA), and *P* < 0.05 was considered statistically significant.

## Results

### Basic characteristics and family history

**Figure 1:**
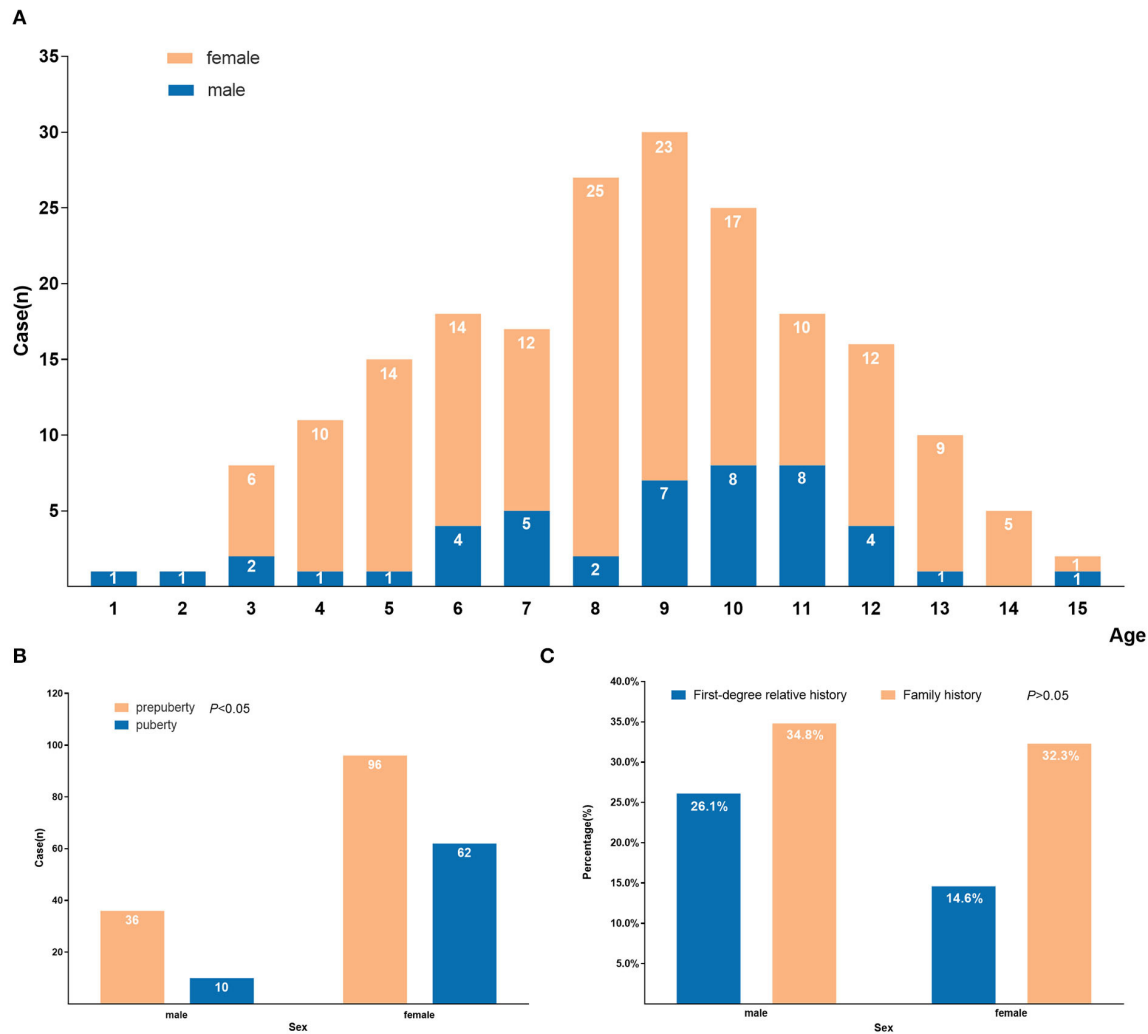
Demographic characteristics of 204 GD children and adolescents. **(A)** Age and sex distribution at first diagnosis of GD; **(B)** Distribution of symptom occurrence during prepuberty and puberty by sex of GD; **(C)** Distribution of positive family history by sex of GD.

Our study enrolled 204 newly diagnosed GD cases, including 158 females and 46 males, and the sex ratio was 3.4:1. The number of different sexes in each age-matched group is shown in Figure 1A. The age at first diagnosis ranged from 1 year and 11 months to 15 years and 6 months, and the average age was 8.9 ± 2.9 years old. One hundred thirty-two cases (64.7%) developed relevant symptoms before puberty, and 72 (35.3%) cases had symptoms during puberty. The sex ratio of the 72 pubertal cases was 62:10 (female: male), and the percentage of pubertal cases was higher in females (39.2%) than males (21.7%) (*P* < 0.05) (Figure 1B). Sixty-seven cases (33.5%, 67/200) had a family history of thyroid disease, with 17.5% (35/200) having a first-degree relative history (12 males and 23 females). The positive rate of boys with a family history was 34.8% (16/46), with 26.1% (12/46) having a first-degree family history. The positive rate of girls with a family history was 32.3% (51/158), with 14.6% (23/158) having a first-level family history. There was no significant difference between the boys and girls in the positive rate of family history (*P* > 0.05) (Figure 1C).

### Symptoms and signs at first-time diagnosis

Exophthalmos or neck swelling was the most common chief complaint at first diagnosis, with a total of 128 cases (62.7%). A few patients visited the hospital because of hyperhidrosis (*n* = 21, 10.3%), weight loss (*n* = 23, 11.3%) and palpitations (*n* = 38, 18.6%). With further inquiry, hypermetabolic symptoms were identified in most of the children and adolescents, and the primary symptoms are shown in Table 1. Some children and adolescents developed chest distress (*n* = 26, 12.7%), polydipsia (*n* = 13, 6.4%), Diuresis (*n* = 8, 3.9%), dizziness (*n* = 12, 5.9%), headache (*n* = 5, 2.5%), sleep disorder (*n* = 7, 3.4%), irregular menstruation (*n* = 2, 1.0%), vomiting (*n* = 3, 1.5%), fainting (*n* = 2, 1.0%) and other symptoms. Fourteen cases (6.9%) visited the hospital because of abnormal thyroid function or rapid heart rate discovered during physical examination. One case reported “right chest pain for 1 week and aggravated for 1 day”, and another case reported “fast growth for half of year with vaginal bleeding for 5 days”. The chief complaints in two cases were “low blood sugar levels for 1 day” and “irregular menstruation for 1 year with neck swelling for 2 months.” Some concomitant diseases were detected at the first diagnosis, such as respiratory tract infection, vitiligo, and Turner's syndrome.

**Table 1:** Retrospective description of symptoms, signs and concomitant diseases after diagnosis of GD.

**Symptoms**	***N***	**Percentage**	**Symptoms**	***N***	**Percentage**
**Hypermetabolic symptoms**			**Non-hypermetabolic symptoms**		
Hyperhidrosis	150	73.5%	Chest distress	26	12.7%
Tremor	130	63.7%	Polydipsia	13	6.4%
Heat intolerance	123	60.3%	Dizziness	12	5.9%
Hyperphagia	117	57.4%	Diuresis	8	3.9%
Irritability	112	54.9%	Sleep disorder	7	3.4%
Poor concentration	100	49.0%	Headache	5	2.5%
Palpitations	88	43.1%	Vomit	3	1.5%
Hyperactivity	85	41.7%	Faint	2	1.0%
Weight loss or no weight gain	85	41.7%	Irregular menstruation	2	1.0%
Decline in academic performance	61	29.9%	Concomitant diseases		
Diarrhea	52	25.5%	Respiratory tract infection	36	17.6%
Weakness	45	22.1%	Vulvitis	1	0.5%
Growth acceleration	6	2.9%	Scoliosis and right kidney absence	1	0.5%
Common symptoms and signs			Vitiligo	1	0.5%
Goiter	193	94.6%	SLE ^a^	1	0.5%
Neck swelling	166	81.4%	Atelencephalia	1	0.5%
Exophthalmos	140	68.6%	Turner's syndrome	1	0.5%

a Systemic Lupus Erythematosus.

### Goiter and exophthalmos

Among the 204 cases diagnosed with GD, 193 cases (94.6%) developed goiter, with 14.0% (27/193) having level I swelling, 79.8% (154/193) having level II swelling, and 6.2% (12/193) having level III swelling. The levels of free triiodothyronine (FT3), FT4 and TPOAb positively correlated with the degree of goiter (*P* < 0.05) (Table 2). There was no statistically significant difference in the sex distribution of different degree of goiter (*P* > 0.05) (Table 3). There were 140 cases (68.6%) of exophthalmos, of which 78.6% (110/140) were simple exophthalmos, and 21.4% (30/140) were infiltrative exophthalmos. The levels of FT3 and FT4 were positively correlated with the degree of exophthalmos (*P* < 0.05) (Table 2). There was no statistically significant difference in the sex distribution of different degrees of exophthalmos (*P* > 0.05) (Table 3).

**Table 2:** The correlations between thyroid function and degree of goiter and exophthalmos.

**Variables^b^**	**goiter**		**exophthalmos**	
	***r*** **^a^**	***P***	***r*** **^a^**	***P***
FT3 (pmol/L)	0.215	**0.002** ^c^	0.249	**<0.001** ^c^
FT4 (pmol/L)	0.202	**0.004** ^c^	0.29	**<0.001** ^c^
TSH (uIU/ml)	−0.026	0.711	−0.065	0.354
TRAB (IU/ml)	0.109	0.122	0.121	0.085
TPOAb (IU/ml)	0.165	**0.026** ^c^	−0.047	0.533
TGAb (IU/ml)	0.063	0.375	−0.111	0.116

a Spearman Rank Correlation Analysis was used.

b FT3, Free triiodothyronine; FT4, Free thyroxine; TSH, Thyroid stimulating hormone; TRAb, thyrotropin receptor antibody; TPOAb, Thyroid peroxidase antibody; TGAb, Thyroglobulin antibody.

c Statistically significant results (*P* < 0.05) are in bold.

**Table 3:** Goiter and exophthalmos between different sexes.

	**Classification**	**Male (N, %)**	**Female (N, %)**	**Chi-square test**	***P***
Goiter	0	4 (8.7%)	7 (4.4%)	2.786	0.426
	I	7 (15.2%)	20 (12.7%)		
	II	34 (73.9%)	120 (75.9%)		
	III	1 (2.2%)	11 (7.0%)		
Exophthalmos	Non	16 (34.8%)	48 (30.4%)	0.817	0.665
	Simple	25 (54.3%)	85 (53.8%)		
	Infiltrative	5 (10.9%)	25 (15.8%)		

Data were presented as N (%).

### Thyroid function

Among the 204 GD cases, 14 (6.9%) cases had mild hyperthyroidism, 39 (19.1%) cases had moderate hyperthyroidism, and 151 (74.0%) cases had severe hyperthyroidism. The positive rate of TRAb was 99.5% (203/204), with 38 (18.6%) cases having significantly elevated values (>40 IU/L). The positive rate of TGAb was 64.2% (129/201), with 8 (4.0%) cases having significantly elevated values (>4,000 IU/ml). The positive rate of TPOAb was 79.0% (143/181), with 17 (9.4%) cases having significantly elevated values (>600 IU/ml). Comparisons of these variables between different sexes revealed no significant differences, except for the level of FT3 (*P* < 0.05) (Table 4).

**Table 4:** Comparison of thyroid hormone and thyroid antibodies levels between different sexes in GD.

	**Range of reference value**	**Female** ***N =*** **158**	**Male *N =* 46**	***P*** **^a^**
FT3	2.8~7.11 pmol/L	33.01 (22.14,43.07)	26.84 (20.38,35.80)	**0.030** ^b^
FT4	12.1~22 nmol/L	84.82 (54.54,100.00)	69.64 (49.40,98.94)	0.055
TSH	0.2 5 uIU/ml	0.005 (0.005,0.006)	0.005 (0.005,0.006)	0.604
TRAb	<1.75 IU/L	19.82 (10.10,35.30)	15.65 (5.80,34.69)	0.249
TPOAb	≤ 34 IU/ml	167.90 (37.63,343.00)	130.60 (56.03,326.95)	0.907
TGAb	0~115 IU/L	347.90 (27.03,843.80)	281.90 (51.65,677.75)	0.501

a Wilcoxon rank sum test was used.

Data were presented as Median (IQR).

b Statistically significant results (*P* < 0.05) are in bold.

### Hemogram and liver function

Abnormal routine blood results were recorded in 14.2% of cases (29/204), with 10 (4.9%) cases having leukopenia, 18 (8.8%) cases having neutropenia, 15 (7.4%) cases having mild anemia, 1 (0.5%) case having agranulocytosis, and 1 (0.5%) case having mild thrombocytopenia during the early stage of the disease. The liver function indices included alanine aminotransferase (ALT), aspartate aminotransferase (AST), alkaline phosphatase (ALP), gamma-glutamyl transferase (γ-GT), total bilirubin (TBIL), direct bilirubin (DBIL), and indirect bilirubin (IBIL), and abnormal results were recorded in 17.8, 7.4, 7.4, 10.9, 9.9, 5.9, and 5.0% of cases, respectively. The overall rate of abnormal liver function was 26.5% (54/204).

## Discussion

GD is an autoimmune disease that is characterized by a series of hypermetabolic symptoms caused by the interaction of TRAb antibody and thyroid-stimulating hormone receptors (TSHR), which promotes the compounding and secretion of thyroid hormones (25). The average age of onset in present study was 8.9 ± 2.9 years old, and the sex ratio was ~3.4:1 (female: male). Females accounted for most patients, and the percentage of pubertal cases was higher in females than males. This result is consistent with current knowledge that GD is more common in peri-pubertal and pubertal females. Previous studies suggested that sex hormones significantly influenced autoimmune diseases, and young females generally have stronger inflammatory responses than males. This relationship may be due to the effects of estrogen on regulatory T cells *via* its receptor (Erα), which stimulates the immune system. Androgens generally play an immunoprotective role in this reaction (26, 27). It may also be related to ectopia or deletion of the X chromosome (28).

GD exhibits an obvious family aggregation, which indicates that genetic factors play an important role in its onset and development. Studies of GD twins showed that genetic factors contributed 70 to 80% to the development of GD (29). Previous studies found that the positive rate of thyroid disease in family history ranged from 30 to 50% in children and adolescents with GD (8, 30), and the positive rate in family history in our study was 33.5%, which is consistent with previous findings. For children and adolescents with a family history, parents should pay attention to the early symptoms and signs of hyperthyroidism to avoid a delay in treatment.

GD patients primarily showed hypermetabolic syndrome. Adults generally visit the hospital due to symptoms such as hyperhidrosis, heat intolerance, hyperphagia, weight loss, hand tremors, and palpitations (23). However, only a few patients came to the hospital because of these symptoms in our study, and most cases came to the hospital due to obvious neck swelling or protruding eyes discovered by their parents. The time interval between the development of symptoms and the visit to a doctor varied and ranged from a few days to several years. We found that most of the cases already had symptoms of hypermetabolism before visiting the doctor, including heat intolerance, hyperhidrosis, hyperphagia, tremor, irritability, palpitations, weight loss or no weight gain, and hyperactivity, which are similar symptoms to adults. However, we also identified two symptoms specific to pediatric patients, namely poor concentration (*n* = 100, 49%) and a decline in academic performance (*n* = 61, 29.9%). Most of the cases had obvious symptoms and abnormal laboratory indices when they visited a doctor, but a lack of self-awareness and parental inattention to early symptoms led to delays in treatment. Therefore, parents should remain vigilant when their children have unexplained weight loss, a decrease in grades, emotional changes, and other abnormalities. Even children and adolescents without neck swelling or exophthalmos should go to the hospital for early detection and treatment to reduce the damage to growth and development. A few cases in our study were initially diagnosed with atypical symptoms, including headache, dizziness, weakness, sleep disorder, and vomiting. Zhu et al. (9) also reported 6 cases of hyperthyroidism in children with special manifestations, such as diuresis, diarrhea, and growth acceleration, who were initially misdiagnosed as neurogenic frequent micturition and myocarditis. Because of the variety of GD manifestations in children and adolescents, clinicians should carefully inquire about medical histories and pay attention to thyroid function tests and physical examinations to reduce misdiagnoses.

Exophthalmos and goiter are the most common signs of GD. The etiology of Graves ophthalmopathy (GO) has not been fully established, it is likely caused by autoimmune reactions due to the orbital distribution of TRAb. The incidence of GO in children and adolescents is similar to adults (31), but the severity is milder. This population primarily exhibits exophthalmos and soft tissue involvement with rare involvement of the ocular muscles and optic nerve. A total of 140 (68.6%) cases developed exophthalmos in the present study, and 21.4% (*n* = 30) of these cases were infiltrative exophthalmos that manifested as gaze, convergence insufficiency, eyelid swelling and lagophthalmos. No severe cases of keratopathy or restrictive strabismus were identified. Holt et al. (31) mentioned that the severity of exophthalmos was obviously lower in children and adolescents than adults. Smoking and aging (32) are important risk factors for the progression of GO in adults, which may explain why GO in children and adolescents is generally less serious. The morbidity of goiter was higher in children and adolescents with GD compared to adult patients, which was attributed to the stimulatory effect of TSAb and thyroid growth-stimulating immunoglobulins (TGIs) on the thyroid. A total of 94.6% of cases developed goiter in the present study, with most cases having level II enlargement, which is consistent with the results of Jiang Xiaoyu (29). Females accounted for 78.2% (*n* = 151) of goiter cases, and males accounted for only 21.8% (*n* = 42), which suggests that females are more likely to develop goiter. Similar findings were identified in adults (33). The current study demonstrated a correlation between the severity of hyperthyroidism and the degree of exophthalmos and goiter. More obvious exophthalmos and goiter indicated more severe hyperthyroidism in children and adolescents.

GD is primarily caused by the combined effects of TRAb and TSHR, which stimulate TH synthesis (34). TRAb is an IgG antibody produced by thyroid B-infiltrating lymphocytes, and it is divided into 3 types based on different functions (35): (1) TSAb have a TSH-like effect and cause excessive secretion of TH after combination with TSHR; (2) Thyroid-stimulation blocking antibody (TSBAb) combines with TSHR to block the function of TSH, which leads to reduced secretion of TH; and (3) TGI stimulates goiter but has no effect on thyroid function. Previous research (36) suggested that ATID patients with TSAb and TSBAb positivity have hyperthyroidism when TSAb plays a leading role and hypothyroidism otherwise. TRAb was tested in all patients at our center. Zophel (37) found that the positive rate of TRAb in GD children was ~60–90%. The positive rate of TRAb was 99.5% in our study, which is roughly consistent with a previous study. TRAb may be used for auxiliary diagnosis because of its high positive rate in children and adolescents with GD. However, the possible conversion between the TSAb and TSBAb makes regular testing of thyroid function and antibodies necessary during the diagnosis and treatment of GD.

TPOAb and TGAb have destructive effects on the thyroid (34). Previous studies (38) showed that both types of antibodies were detectable in nearly half of GD patients. The positive rates of TGAb and TPOAb in our study were 64.2% (129/201) and 79.0% (143/181), respectively. Yuan et al. (39) showed no significant difference in the positive rates of TGAb and TPOAb between the GD group and the Hashimoto thyroiditis (HT) group in an adult population. However, they found that the antibodies in the GD group may have a weaker ability to cause inflammation than in the HT group and detecting IgG subtypes of TPOAb and TGAb may be helpful for the differential diagnosis of GD and HT. Subtypes of TPOAb and TGAb were not measured in the present study, hence, further research is needed.

Studies in adult patients with GD indicated that anti-thyroid drugs and GD per se may cause hematological abnormalities, including anemia, leukopenia, thrombocytopenia, and pancytopenia (19, 40, 41). There have been few relevant studies in children and adolescents. Our study found that the total incidence of abnormal hemogram at initial diagnosis in our center was 14.2% and primarily involved neutropenia, leukopenia, and mild anemia. The results of routine blood tests should receive more attention.

According to previous reports (13, 17, 42), the incidence of liver dysfunction in patients with hyperthyroidism is common. Wang et al. found that age, a long history of GD, and higher FT3 and TRAb concentrations were likely to be associated with liver dysfunction. The liver function indices in the present study included ALT, AST, ALP, γ-GT, TBIL, DBIL and IBIL. The overall incidence of liver dysfunction in the present study was 26.5%, which is generally consistent with previous research. The pathogenesis of liver dysfunction has not been defined, but we propose that it may be related to the following factors (18): (1) direct damage from excessive TH, (2) inhibited activity of glucuronyl transferase (UGT) in the liver, (3) disturbed excretion of serum bilirubin, (4) development of cholestatic jaundice, (5) relative lack of oxygen in the liver under a hypermetabolic condition, (6) thyrotoxicosis affecting the activities of various enzymes in the liver, (7) liver damage caused by autoimmune reactions. Due to the high proportion of liver dysfunction in children and adolescents, liver function examination is recommended during a preliminary diagnosis.

The current study reviewed the characteristics of the GD profile of children and adolescents in our hospital from 2013 to 2019 and collected and described their symptoms, signs, and laboratory blood biochemical indices. The present study identified specific symptoms in children and adolescents, such as poor concentration and a decline in academic performance, in addition to the typical symptoms observed in adults. The atypical symptoms and signs were summarized. These findings may help clinicians with decision-making and reduce missed and misdiagnosis of GD. However, the present study also has shortcomings that need improvement. As a pediatric specialty hospital, we may have lost some of the older teenagers who completed their initial diagnosis or treatment in adult hospitals. The missing sample may affect our summarization of the characteristics of GD in children and adolescents in our center. Because the sample size of this single-center retrospective study was limited, our conclusions should be verified in a larger sample.

## Conclusion

GD was more common in females, and physiological changes during puberty may have a greater impact on the morbidity of GD in females. Routine blood and liver function tests are recommended at the time of initial diagnosis. It is necessary to increase public awareness of the early symptoms of GD to avoid delays in treatment. Further studies with large samples should be performed to reveal the specific mechanisms of GD-related liver dysfunction and provide treatment strategies.

## Data availability statement

The raw data supporting the conclusions of this article will be made available by the authors, without undue reservation.

## Ethics statement

This study involving human data was reviewed and approved by the Medical Ethics Committee of the Children's Hospital of Nanjing Medical University (No: 2022011003-1). Written informed consent to participate in the study was not required in accordance with the national legislation and the institutional requirements.

## Author contributions

HX checked the final data and result of analyses, drafted, and polished the initial manuscript. DC and JZ collected and cleaned up data, carried out the initial analyses, and drafted the initial manuscript. RY checks the final data and result of analyses. XW and WG conceptualized and designed the study, coordinated, supervised data collection, check the final data, result of analyses, and critically reviewed the manuscript for important intellectual content. All authors approved the final manuscript as submitted and agree to be accountable for all aspects of the work.

## Funding

This work was supported by the National Natural Science Foundation (82173536).

## Conflict of interest

The authors declare that the research was conducted in the absence of any commercial or financial relationships that could be construed as a potential conflict of interest.

## Publisher's note

All claims expressed in this article are solely those of the authors and do not necessarily represent those of their affiliated organizations, or those of the publisher, the editors and the reviewers. Any product that may be evaluated in this article, or claim that may be made by its manufacturer, is not guaranteed or endorsed by the publisher.
